# Homogenization of the vertically stacked medium frequency magnetic metamaterials with multi-turn resonators

**DOI:** 10.1038/s41598-022-24809-y

**Published:** 2022-11-25

**Authors:** Adam Steckiewicz

**Affiliations:** Bialystok University of Technology, Faculty of Electrical Engineering, 15-351 Białystok, Poland

**Keywords:** Computational methods, Characterization and analytical techniques, Electrical and electronic engineering

## Abstract

The paper presents a homogenization method of the magnetic metamaterials, made of perpendicularly oriented resonators consisting of multi-turn planar coils. A resulting composite, in the form of parallel stripes with metamaterial cells, exhibits extraordinary properties in the medium frequency magnetic field, such as zero permeability. To identify an effective permeability of this metamaterial, two models were presented, i.e., a three-dimensional numerical model with current sheet approximation as well as Lorentz oscillator model, where individual coefficients are based on the lumped circuit parameters and directly related with a geometry of the unit cell. The accuracy of the second approach is improved by taking into account mutual inductances in a metamaterial grid. Then, a comparison is made with numerical model results to show adequacy of the adopted analytical attempt, and properties of this type of metamaterial are discussed. It is shown that discussed metamaterial structure can achieve negative permeability as well as its values, at identical resonant frequency, are dependent on number of turns of the planar coil.

## Introduction

Metamaterials were introduced at the end of the twentieth century and gained great attention in the scientific and industrial environment. Due to an ability to exhibit unordinary properties, for instance a zero or negative permittivity and permeability^1–4^, a potential interest of creating new functional structures had risen. The synthesis of superlenses^5,6^, cloaks^7–9^, new types of antennas^2,10^ and absorbers^11–13^ and devices for medical diagnosis^14,15^ are some of many considered applications. Recent advances in terahertz metamaterials also show an accelerating progress of tunable metastructures^16,17^, where a resonance wavelength can be tuned using appropriate external fields. A dynamic development of metamaterials in a high frequency electromagnetic field also encouraged scientists to analyze stationary as well as low frequency fields, and resulted in Laplacian metamaterials^18,19^. The scope of usage had extended to extraordinary devices, such as invisibility shells for sensors^7^, artificial wormholes^20^, magnetic hoses^21^, lenses increasing the resolution of magnetic resonance imaging^22–24^ and effective shielding of a low frequency magnetic field^25,26^. Nevertheless, some of these were intended to use in the stationary magnetic field, e.g., magnetic hose, through the utilization of anisotropic structures: layers of ferromagnetic material and superconductor with nearly zero permeability^19^. Still, it may be possible to mimic these abilities in alternating magnetic field at a specific frequency, if metamaterials characterized by identical properties would be synthesized. To effectively design such composite materials, a fast and accurate homogenization method to estimate their effective properties would be strongly desired.

Despite the low and high frequency structures, the medium frequency magnetic field metamaterials were discussed as well. Their topology is similar to the high frequency metamaterials, for example the split-ring resonators^27,28^, hence unit cells consist of an inductive element (in this case a planar coil) made of a conductive material and connected with capacitance (parasitic, or in a form of the lumped capacitor), which are then arranged periodically to form the structure with effective properties. Aside from the classical horizontally stacked unit cells, some examples of vertically stacked resonators were shown^1,11,29–31^, to create a composite possessing identical outer dimensions but able to interact with other component of an external field. In most cases, applications in the relatively high frequencies were considered, e.g., in MHz range^32–35^. Including widely developed stationary field meta-structures a peculiar gap, covering frequencies higher than several kHz but less than MHz band, in the used frequency spectrum of magnetic metamaterials can be observed.

It is important, since promising application of the medium frequency metamaterials was recently found in the wireless power transfer (WPT) systems^36–38^. The metamaterial can act as the device enhancing a magnetic coupling between transmitting and receiving coil^38–40^, which led to an increase of an efficiency of the WPT system^34,37,38^. There are several different types of metamaterials used for this purpose, where many of them were based on the printed circuit board planar coils with on-chip capacitors^32–34,36^. They are also varying in terms of positioning with relation to transmitting and receiving coil, e.g., parallel or perpendicularly oriented in-between them, behind these coils or in front/behind one of them^35,39,40^. This shows that both resonators and their position in space has a crucial impact on WPT system performance, yet properties of the medium frequency magnetic field metamaterials are not well known, and multi-turn resonators were not fully characterized either.

The extraction of medium frequency metamaterials effective properties is the most important part of their analysis. The homogenization of the unit cell can be performed using several methods, such as field averaging^41^, Bloch wave^42–44^ or scattering parameters (S-parameters)^13,45^. These methods, however, are based on a spatial field distribution, which is obtained predominantly from three-dimensional (3D) numerical model solved in a frequency domain. Since the number of degrees of freedom (NDOF) quickly becomes large in 3D models, the demand for computing memory rises sharply, putting pressure on computer memory, and the calculation time is also prolonged. To avoid disadvantages of numerical approach the analytical methods were used^28,46^ to estimate effective properties. Unfortunately, due to a complex derivation of the final formulae they are limited to the simplest or the best-known geometries of meta-cells. Techniques combining general solution of the analytical formulation with numerically (or experimentally) identified coefficients were also introduced. For example, the Lorentz oscillator model was used in^47^ and the quality factor and resonant frequency were found based on a modified 3D numerical model. To the best of the author's knowledge, no purely analytical solution was presented for the medium frequency metamaterials made of planar coils, especially of a multi-turn type, with resonators stacked vertically in space.

In this article such technique of homogenization was presented. The main focus was put on numerical modeling and calculations, but mostly on determining formulae and procedure for analyzing discussed composite material by analytical approach. Accordingly, the vertically stacked stripes of resonators, forming metamaterial intended for the medium frequency bandwidth, were analyzed numerically (3D model) and by the Lorentz oscillator model. The novelty included in the second approach can be found in taking into account magnetic couplings in the periodic grid and derived equations, where all coefficients of the Lorentz model were identified and directly related with the geometry of the unit cell and a type of planar coil. This allows estimating permeability of metamaterial without numerical model or experimentally identified coefficients, reducing computational requirements. While the numerical approaches were not able to find properties of each individual unit cell in a finite grid, the presented analytical model opens this possibility. An analysis of the effective permeability was performed for different resonant frequencies, distances between layers and filling factor of the resonator. Both models were compared and the results as well as accuracy of the proposed analytical model were discussed.

## Analyzed structure

**Figure 1:**
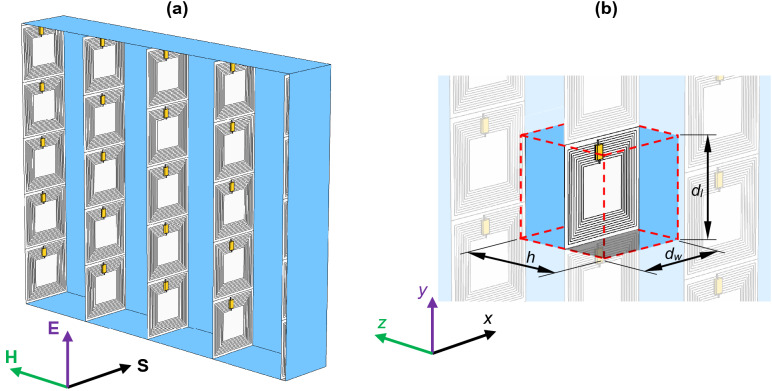
(**a**) Considered metamaterial composite consisting of an array of vertically stacked planar coils connected with lumped capacitors. Resonators are perpendicularly oriented with respect to magnetic field **H** of the incident plane wave **S**. (**b**) Unit cell of the metamaterial structure with a length *d*_*l*_, width *d*_*w*_ and height *h*.

The medium frequency metamaterial is intended to operate at frequencies higher than 10 kHz but less than 10 MHz. In this bandwidth it is possible to create a metamaterial unit cell consisting of the periodic grid of resonators. These resonators are constructed of planar coils, interacting with external magnetic field, and a capacitance to achieve a resonance at some specific resonant frequency *f*_0_. Typical approach of realizing this metamaterial is to form layers of a rectangular 2D periodic grid of horizontally stacked resonators. Yet, another possibility is to orient these unit cells perpendicularly and utilize only one column of the periodic grid. As the result, instead of 2D array, a pack of many stripes with linearly organized resonators is used to build the composite, as shown in Fig. 1a. An elementary unit cell, presented in Fig. 1b, has centrally located planar coil connected in series with a lumped capacitor. The cell has width *d*_*w*_, length *d*_*l*_ and the distance between stripes is *h*. Hence, the thickness of the metamaterial is equal to the width of unit cell.

**Figure 2:**
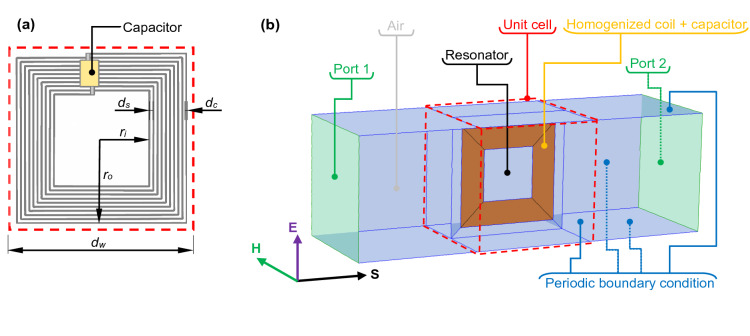
(**a**) A view on the resonator wounded using thin wires with diameter *d*_*c*_ and separation *d*_*s*_. (**b**) The 3D numerical model of the metamaterial (unit cell) with boundary conditions and homogenized planar coil.

The resonator is made in a form of multi-turn coil with attached lumped capacitor, as shown in Fig. 2a. Although in Figs. 1 and 2 the square planar coils were presented, the shape of coil can be changed to, e.g., circular or octagonal and even deformed. For example, the width of cells and, simultaneously, the thickness of the metamaterial may be reduced utilizing planar coil with rectangular shape, where *d*_*w*_ ≠ *d*_*l*_. The main idea is to wound the turns of any type of the planar coil using ultra-thin wire, where a diameter of conductor is *d*_*c*_, separation between wounds is *d*_*s*_ and the number of all turns is *n*. When the outer size of the planar coil is *r*_*o*_, then the inner resection will be expressed as

1 $$r_{i} = r_{o} - nd_{c} - \left( {n - 1} \right)d_{s} .$$

An application of ultra-thin wire has two main advantages: firstly, many turns can be wounded in an area restricted by outer size *r*_*o*_ which will result in a higher self-inductance and secondly, if the diameter of the conducting part is less than skin depth, than the skin effect can be omitted in a mathematical model. On the other hand, the lesser diameter will result in a smaller cross-section area of the wire and hence a greater equivalent resistance of the coil.

## Numerical model

The numerical model of the presented metamaterial structure can be reduced to a single cell. This approach simplifies the modeling task and decreases NDOF with respect to the model of the entire composite material. The considered three-dimensional model, presented in Fig. 2b, is a cuboid consisting (in the central part) of the unit cell with homogenized multi-turn resonator as well as two parts of an air (ambience) and boundary conditions. To mimic the infinite periodic arrangement of vertically stacked stripes, the periodic boundary condition is assigned to all outer surfaces, except for bases of the cuboid where lumped ports are imposed. Port 1 is a source of an external magnetic field with the only vector component directed perpendicularly to the surface of the resonator, so in this case **H** = [0 *H*_*y*_ 0]. Port 2 does not excite any external field since it acts as a perfectly absorbing surface which imitates infinite space behind the metamaterial.

The distribution of the magnetic field in a frequency domain can be solved using magnetic vector potential **A** = [*A*_*x*_ *A*_*y*_ *A*_*z*_], defined as *μ*_*r*_*μ*_0_**H** = ∇ × **A**, and formulation of the phenomena through the Helmholtz equation^48^

2 $$\nabla \times \left( {\frac{1}{{\mu_{r} \mu_{0} }}\nabla \times {\mathbf{A}}} \right) - {\text{j}}\omega \sigma {\mathbf{A}} = 0,$$

where: *μ*_*r*_ is the relative permeability; *μ*_0_ is the permeability of air (H/m); *ω* is the angular frequency (rad/s); *σ* is the electrical conductivity (S/m). The solution of the Eq. (2) in 3D space can be obtained using, e.g., the finite element method (FEM)^4^.

A crucial part of the model is a homogenized multi-turn coil. Without any homogenization the complex structure, shown in Fig. 2a, would have to be recreated in the model, along with the thin wires (*d*_*c*_) and even thinner separations (*d*_*s*_). From the modeling point of view, it is troublesome task leading to the greater NDOF, since each thin part must be reconstructed by extremely fine mesh. As the result, the model may not be solved, or it will require long computation time. A solution to this problem is a current sheet approximation^49,50^ or an equivalent foil approach^51^. In the software used for the computations of the introduced metamaterial it is known as the Homogenized multi-turn boundary condition^52^. This approach implements a homogenized model of a coil consisting of many conducting wires or printed paths, where the number of wires and their cross-section area are taken into account. Due to this homogenization, coils containing many wires can be modeled as the negligibly thin conducting sheets.

The magnetic field distribution will be determined based on the magnetic vector potential. The distribution of magnetic field on the surfaces of Port 1 and Port 2 is the most important since magnetic field values are used to find the scattering parameters *S*_11_ and *S*_21_^45^. These parameters and equations allow identifying the effective permeability of the metamaterial^13^.

## Lorentz oscillator model

An alternative for numerical modeling of metamaterials is an analytical approach, based on the general formulation of the phenomena occurring in the meta-cell. In various works^2,28,46,47,53^ the formula for complex effective permeability *μ*_*eff*_ = *μ*_*Re*_ + j*μ*_*Im*_ was studied

3 $$\mu_{eff} = 1 - \frac{F}{{1 - \frac{{f_{0}^{2} }}{{f^{2} }} + {\text{j}}\frac{{f_{0} }}{fQ}}},$$

where: *f*_0_ is the resonant frequency (Hz); *F* is the fractional volume factor; *Q* is the quality factor. These three main parameters of the function expressed by Eq. (3) must be found to describe the properties of the unit cell. The fractional volume factor is^47^

4 $$F = \frac{{\mu_{0} \left( {\sum\limits_{i = 1}^{n} {S_{i} } } \right)^{2} }}{{L_{eff} V}},$$

where: *L*_*eff*_ is the effective inductance of the unit cell (H); *S*_*i*_ is the area of *i*-th loop of the coil (m^2^); *V* is the volume of the cell (m^3^), which is

5 $$V = hd_{w} d_{l} .$$

**Figure 3:**
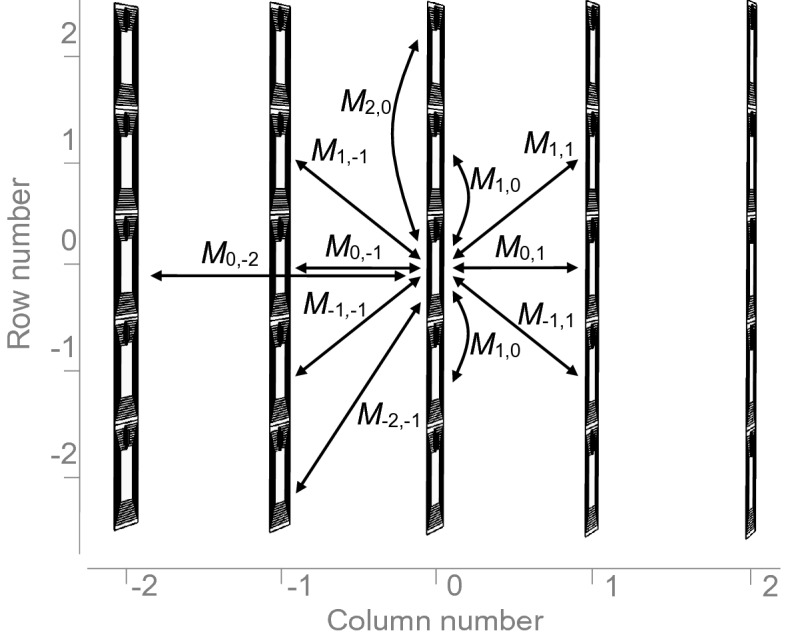
Front view on the periodic structure of resonators with indicated mutual inductances between considered unit cell and other cells in the array.

The analyzed unit cell is in a specific position within the metamaterial structure and surrounded by the other cells, as shown in Fig. 3. The magnetic coupling between this unit cell and each resonator appears and affects the effective magnetic properties. Similar situation was previously considered in the periodic arrays of wireless power transfer systems^54^ and it was found that inductive magnetic couplings are directly affecting the inductance of a coil by adding to its self-inductance all mutual inductances between considered inductor and all the others. Hence, at this point it will be assumed that effective inductance of the unit cell can be expressed as

6 $$L_{eff} = L_{self} + M = L_{self} + \sum\limits_{a} {\sum\limits_{b} {M_{a,b} } } ,$$

where: *L*_*self*_ is the self-inductance of the planar coil (H); *M* is the sum of mutual inductances (H); *a*, *b* is the row and column number in the periodic grid, *a* ≠ 0 ˄ *b* ≠ 0; *M*_*a,b*_ is the mutual inductance between an arbitrary unit cell and the cell in *a*-th row and *b*-th column (H). The mutual inductance *M*_*a,b*_ is^55^

7 $$M_{a,b} = \rho \frac{{\mu_{0} g^{2} }}{4\pi }\int\limits_{{\Phi_{{\text{i}}} }}^{{\Phi_{{\text{o}}} }} {\int\limits_{{\Phi_{{\text{i}}} }}^{{\Phi_{{\text{o}}} }} {\frac{{\left( {1 + \varphi_{1} \varphi_{2} } \right)\cos \left( {\varphi_{2} - \varphi_{1} } \right) - \left( {\varphi_{2} - \varphi_{1} } \right)\sin \left( {\varphi_{2} - \varphi_{1} } \right)}}{{\sqrt {\left( {\left| b \right|h} \right)^{2} + \left( {\left| a \right|d_{l} + g\varphi_{2} \cos \varphi_{2} - g\varphi_{1} \cos \varphi_{1} } \right)^{2} + \left( {g\varphi_{2} \sin \varphi_{2} - g\varphi_{1} \sin \varphi_{1} } \right)^{2} } }}{\text{d}}\varphi_{1} {\kern 1pt} {\text{d}}\varphi_{2} } } ,$$

where: *g* = (*d*_*c*_ + *d*_*s*_)/(2π); Φ_*i*_ = *r*_*i*_/*g*, Φ_*o*_ = *r*_*o*_/*g*; *φ*_1_, *φ*_2_ are the elementary angles (rad); *ρ* is the shape factor related with the type of coil, e.g., for circular *ρ* = 1 while for a square *ρ* varies from 1.1 to 1.27^56,57^. After applying the rectangle rule Eq. (7) takes the following form^54^

8 $$M_{a,b} = \rho \frac{{\mu_{0} g^{2} \,}}{4\pi }\sum\limits_{{k_{2} = 1}}^{K} {{\kern 1pt} \sum\limits_{{k_{1} = 1}}^{K} {\frac{{\left( {1 + k_{1} k_{2} \Phi_{K}^{2} } \right)\cos \left( {k_{2} \Phi_{K} - k_{1} \Phi_{K} } \right) - \Phi_{K} \left( {k_{2} - k_{1} } \right)\sin \left( {k_{2} \Phi_{K} - k_{1} \Phi_{K} } \right)}}{{\sqrt {\left( {\left| b \right|h} \right)^{2} + \left( {\left| a \right|d_{l} + gk_{2} \Phi_{K} \cos k_{2} \Phi_{K} - gk_{1} \Phi_{K} \cos k_{1} \Phi_{K} } \right)^{2} + \left( {gk_{2} \Phi_{K} \sin k_{2} \Phi_{K} - gk_{1} \Phi_{K} \sin k_{1} \Phi_{K} } \right)^{2} } }}} } ,$$

where: Φ_*K*_ = (Φo – Φi)/*K* is an integration step; *K* is assumed number of integration subintervals, *K* ≥ *r*_*o*_/*g* and *K* ∈ N.

The self-inductance of a coil can be estimated using formula^58^

9 $$L_{self} = \frac{{c_{1} \mu_{0} d_{avg} n^{2} }}{2}\left[ {\ln \left( {\frac{{c_{2} }}{v}} \right) + c_{3} v + c_{4} v^{2} } \right],$$

where *d*_*avg*_ is a mean diameter, e.g., for a circular or square coil it will be

10 $$d_{avg} = r_{o} + r_{i} ,$$

and *v* is a fill factor

11 $$v = \frac{{r_{o} - r_{i} }}{{r_{o} + r_{i} }},$$

while *c*_1_, *c*_2_, *c*_3_, *c*_4_ are shape coefficients which differs for a square, circular, hexagonal, octagonal or any other shape of a planar coil^58^. The fractional volume in Eq. (4) also requires the sum of areas of each loop of the coil. The area of *i*-th loop is

12 $$S_{i} = \left[ {2r_{o} - \left( {2i - 1} \right)\left( {d_{c} + d_{s} } \right)} \right]^{2} .$$

Assuming that square coils are used to build a metamaterial, the sum of all areas from Eq. (12) may be calculated as

13 $$\sum\limits_{i = 1}^{n} {S_{i} } = \frac{{4r_{o}^{3} - 4\left[ {r_{o} - n\left( {d_{c} + d_{s} } \right)} \right]^{3} - n\left( {d_{c} + d_{s} } \right)^{3} }}{{3\left( {d_{c} + d_{s} } \right)}}.$$

Finally, using Eqs. (5), (6) and (13) the fractional factor can be expressed in a form

14 $$F = \frac{{\mu_{0} \left\{ {\frac{{4r_{o}^{3} - 4\left[ {r_{o} - n\left( {d_{c} + d_{s} } \right)} \right]^{3} - n\left( {d_{c} + d_{s} } \right)^{3} }}{{3\left( {d_{c} + d_{s} } \right)}}} \right\}^{2} }}{{\left( {L_{self} + \sum\limits_{a} {\sum\limits_{b} {M_{a,b} } } } \right)hd_{w} d_{l} }}.$$

From Eqs. (1), (8)–(11) it can be seen that for any type of a planar coil the coefficients *ρ*, *c*_1_–*c*_4_ can be taken from the literature and then, for a given outer size (*r*_*o*_), number of turns (*n*) and used conductor (with diameter *d*_*c*_ and separation *d*_*s*_), the effective inductance from Eq. (6) can be found, and then substituted into Eq. (14) to calculate the fractional factor.

The second parameter is a quality factor (*Q*). A resonator in the unit cell acts as a RLC circuit, hence the quality factor is

15 $$Q = \frac{{2\pi f_{0} L_{eff} }}{R},$$

where *R* is a resistance of the coil defined as

16 $$R = \frac{{l_{total} }}{{\sigma_{c} a_{c} }},$$

where: *l*_*total*_ is the total wire length (m); *σ*_*c*_ is the electrical conductivity of a wire (S/m), *a*_*c*_ is the wire cross-section area (m^2^). The length of the wire can be simply obtained as a product of the number of turns and an average perimeter (half length of the outer edges and edges of the inner resection). Hence, for a square shape planar spiral, wounded by a wire with a circular cross-section, the resistance can be obtained using formula

17 $$R = \frac{{n\left( {\frac{{8r_{o} + 8r_{i} }}{2}} \right)}}{{\sigma_{c} \left( {\pi \frac{{d_{c}^{2} }}{4}} \right)}} = \frac{{16n\left( {r_{o} + r_{i} } \right)}}{{\sigma_{c} \pi d_{c}^{2} }}.$$

For a printed spiral coil with rectangular cross-section and copper path thickness *t*, simply *a*_*c*_ = *d*_*c*_*t* must be substituted in Eq. (16). Finally, the resonant frequency (*f*_0_) of the resonator will be found based on a series RLC circuit resonance condition

18 $$f_{0} = \frac{1}{{2\pi \sqrt {L_{eff} C} }}.$$

If in Eq. (18) the resonant frequency will be imposed, then a capacitance (*C*) can be derived. In further investigations this second approach was applied to show that if mutual inductances *M* would be omitted in Eq. (6), then the resonant frequency will not be properly estimated in Lorentz model, presented in Eq. (3). Nevertheless, the formulae shown above indicates that all parameters in this model are directly related with the unit cell geometry, such as distance between layers (*h*), pitch size (*d*_*l*_), size of the planar coil (*r*_*o*_) and inner resection (*r*_*i*_). As a result, the effective permeability can be estimated without numerical model and much less computational effort (i.e., computer memory and calculation time) must be used. All equations are also valid for coils with any number of turns; both printed and wounded using a wire. Additionally, the extremes of a real part of the Eq. (3) can be now easily found as

19 $$\max \left( {\mu_{{\text{Re}}} } \right) = \frac{{FQ^{2} }}{1 + 2Q} + 1,$$

20 $$\min \left( {\mu_{{\text{Re}}} } \right) = \frac{{FQ^{2} }}{1 - 2Q} + 1,$$

which are determined by the fill and quality factor. Both these factors are dependent on effective inductance and geometry of a coil as well as the quality factor, as shown in Eq. (15), makes them also dependent on a resonant frequency.

## Results and discussion

An exemplary metamaterial was studied to show a validity of the analytical Lorentz oscillator model. The metamaterial with vertically stacked stripes of meta-cells, shown in the previous chapter, was analyzed from a point of view of a single cell located in the center of the infinitely expanding periodic grid of resonators. The square planar coil with outer size *r*_*o*_ = 20 mm was used. Two cases of the distance between layers were examined: *h* = *d*_*w*_ = 44 mm and *h* = *d*_*w*_/2 = 22 mm. Electrical conductivity of a wire (copper) was *σ*_*c*_ = 5.7·10^7^ S/m. In analytical models, the coefficients used for estimating a self-inductance were *c*_1_ = 1.26, *c*_2_ = 2.08, *c*_3_ = 0.14, *c*_4_ = 0.115. For a mutual inductance a shape coefficient was *ρ* = 1.27 and the number of rows and columns was limited to *a*, *b* ∈ < –10; 10 > .

The frequency bandwidth was limited to *f* ∈ < 100 kHz; 1 MHz > . Within this limit five desired resonant frequencies were imposed *f*_*z*_ = {200, 375, 550, 725, 900} kHz. To achieve a resonance in meta-cell a proper capacitor has to be used. Based on Eq. (18) it can be concluded that effective inductance has an impact on a resonant frequency. To show the shift of this frequency, resulting from various *L*_*eff*_ in different approaches (numerical and analytical), the same capacitor should be used. The simplest approach to find its capacitance is to assume identical “basic” inductance for each model, e.g., one of the possibilities is a self-inductance, effortless to find using Eq. (9). Therefore, the lumped capacitance adopted for the models was estimated as

21 $$C = \frac{1}{{\left( {2\pi f_{z} } \right)^{2} L_{self} }}.$$

The resulting geometrical parameters and capacitances connected to planar resonators in the unit cells are presented in Table 1. The numerical model of a meta-cell, presented in Fig. 2b, was solved by FEM. COMSOL Multiphysics 4.3b software was used, where in AC/DC module (Magnetic Fields physics) the frequency domain calculations were performed. The model was complemented by an additional electrical circuit, where an ideal lumped capacitor was connected in series to a homogenized multi-turn inductor.

**Table 1:** Parameters of resonators with outer radius *r*_*o*_ = 20 mm.

*d*_*c*_ (µm)	*d*_*s*_ (µm)	*d*_*w*_ = *d*_*l*_ (mm)	*r*_*i*_ (mm)	*n* (–)	*L*_*self*_ (µH)	*C* (nF)				
						*f*_*z*_ = 200 kHz	*f*_*z*_ = 375 kHz	*f*_*z*_ = 550 kHz	*f*_*z*_ = 725 kHz	*f*_*z*_ = 900 kHz
200	10	44	15	24	43	14.74	4.19	1.95	1.12	0.73
200	10	44	13	33	66	9.52	2.71	1.26	0.72	0.47
200	10	44	11	43	91	6.92	1.97	0.92	0.53	0.34
200	10	44	9	52	111	5.71	1.62	0.75	0.43	0.28
200	10	44	7	62	128	4.95	1.41	0.66	0.38	0.24
200	10	44	5	71	138	4.59	1.31	0.61	0.35	0.23
200	10	44	3	81	143	4.41	1.26	0.58	0.34	0.22

The main parameter varying during tests was the number of turns (*n*) to study the characteristics and properties of metamaterial. More wounds help to improve a self-inductance (*L*_*self*_), but also lead to a greater resistance (*R*), which limits possible values of the real part (*μ*_*Re*_) and increases imaginary part (*μ*_*Im*_). To make the analysis more general, the influence of *n* on the effective permeability (*μ*_*eff*_) was presented in function of fill factor (*v*) from Eq. (11), dependent directly on *n*, and thereby representing the extent to which the available area of a planar coil is occupied by the turns. Abovementioned cases were calculated using three models:

- 3D numerical model with homogenized planar coil (FEM),
- proposed Lorentz oscillator model with mutual inductances *M* (LMM),
- Lorentz model without *M* (LM).

**Figure 4:**
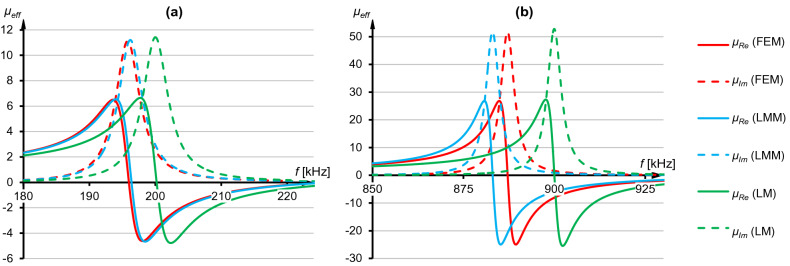
Real and imaginary parts of the effective permeability obtained using three models at *h* = *d*_*w*_, for the specified resonant frequencies: (**a**) *f*_*z*_ = 200 kHz. (**b**) *f*_*z*_ = 900 kHz.

The frequency characteristics for cells at a distance *h* = *d*_*w*_ were shown in Fig. 4. In all cases, a typical shape of *μ*_*Re*_ and *μ*_*Im*_ function was obtained, leading to a dynamic change from positive to negative values of the real part and a maximum value of *μ*_*Im*_ at resonant frequency. In Fig. 4a numerical model (FEM, red lines) has indicated that lower actual resonant frequency (*f*_0_ = 195,7 kHz) was obtained than the one imposed (*f*_*z*_ = 200 kHz), if the capacitance from Eq. (21) was used in a resonator. Nearly identical characteristics were achieved using Lorentz model with a sum of mutual inductances (LMM, blue lines) where actual resonant frequency was *f*_0_ = 196,2 kHz, and the extreme values of the real and imaginary part are consistent with the one from numerical model. However, the analytical model which was not considering magnetic couplings (LM, green lines) preserved unchanged resonant frequency (*f*_0_ = *f*_*z*_). In case of LM the effective inductance is equal to self-inductance, hence frequencies from Eqs. (18) and (21) are the same.

In Fig. 4b it can observed that between FEM and LMM models differences became greater than in Fig. 4a (*f*_0_^FEM^ = 887 kHz and *f*_0_^LMM^ = 883 kHz), yet still smaller than comparing FEM to LM (*f*_0_^FEM^ = 887 kHz and *f*_0_^LM^ = 900 kHz). In this specific instance the resonant frequency shift from LMM was more significant than the one from FEM (*f*_0_^LMM^ < *f*_0_^FEM^), even though numerical model considers all mutual inductances in the grid, hence FEM computed *L*_*eff*_ should be the highest. As a result, according to Eq. (18), *f*_0_^FEM^ always ought to be the lowest. The discrepancy in Fig. 4b is the example of potential issue which may come from the usage of Eq. (7). Numerical solution of this formula produces additional errors, finite number of rows and columns is considered during the calculations and *ρ* may be different (it strongly depends on coil's structure and varies from 1.1 to 4/π). Thus, the calculated *M* is an approximation and, in some cases, can produce higher *L*_*eff*_ in LMM than in FEM. Such situation is not correct and to avoid it *ρ* has to be chosen carefully.

**Figure 5:**
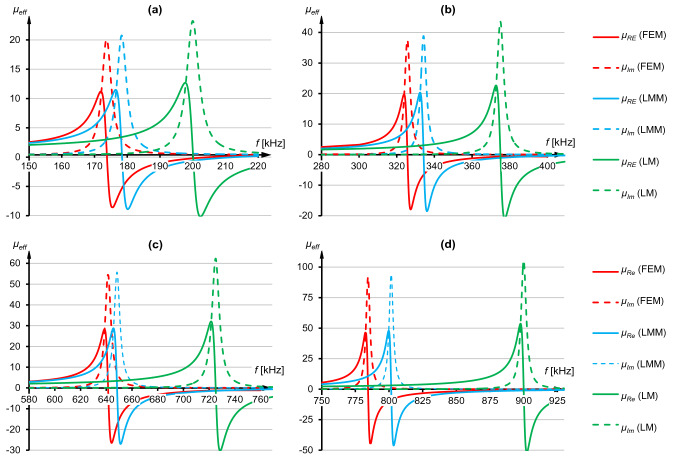
Real and imaginary parts of the effective permeability obtained using three models at *h* = *d*_*w*_/2, for specified resonant frequencies: (**a**) *f*_*z*_ = 200 kHz. (**b**) *f*_*z*_ = 375 kHz. (**c**) *f*_*z*_ = 725 kHz. (**d**) *f*_*z*_ = 900 kHz.

After reducing distance between stripes to *h* = *d*_*w*_/2 the divergences in favor of LMM approach were more significant, which can be seen comparing Figs. 4a and 5a as well as Figs. 4b and 5d. In Fig. 5 it was shown that resonant frequency and values of effective permeability from LMM were closer to FEM results than LM. Differences between the maximum values of real and imaginary parts were also visible, especially comparing FEM with LM (Fig. 5c). For example, in Fig. 5b max(*μ*_*Im*_) from LM was 43.45, while from FEM it was 37.43 and from LMM it was 38.83. This example shows a dependency of both real and imaginary parts on mutual inductances appearing in the metamaterial made of vertically stacked unit cells. Hence, it follows that magnetic couplings in the periodic grid must be taken into account during calculations of the capacitance, to more accurately predict both resonant frequency and values of effective permeability at any frequency.

**Figure 6:**
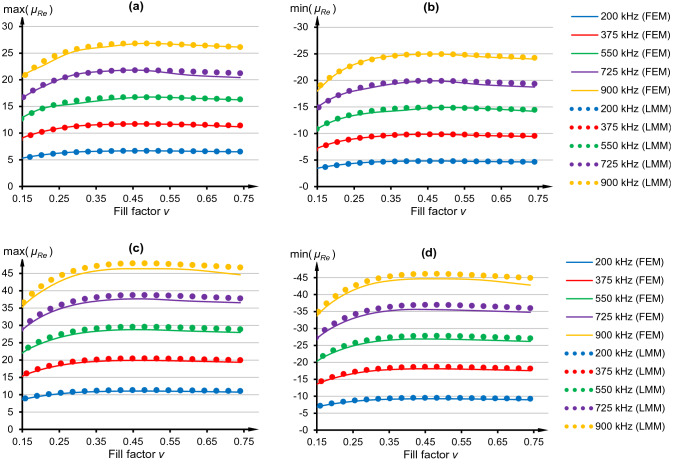
Effective permeability for specified resonant frequencies and two models: (**a**) Maximum value of the real part at *h* = *d*_*w*_. (**b**) Minimum value of the real part at *h* = *d*_*w*_. (**c**) Maximum value of the real part at *h* = *d*_*w*_/2. (**d**) Minimum value of the real part at *h* = *d*_*w*_/2.

In Fig. 6 extremes of the real part were shown and LMM approach was again compared with FEM results. One can conclude that for a fill factor (*v*) increasing from 0 to 0.45, which was caused by the larger number of turns (*n*), maximum and minimum values of the effective permeability were increasing. For the higher fill factors a decrease of these values occurred, which was mainly observed for the highest resonant frequency (900 kHz). Metamaterial exhibited paramagnetic and ferromagnetic behavior since max(*μ*_*Re*_) exceeds 26 at *h* = *d*_*w*_, as shown in Fig. 6a, and 46 at *h* = *d*_*w*_/2 (Fig. 6c). Moreover, the negative permeability was obtained and reached even −25 at *h* = *d*_*w*_, as shown in Fig. 6b, and −45 at *h* = *d*_*w*_/2 (Fig. 6d). The most interesting observation is that optimal values of max(*μ*_*Re*_) or min(*μ*_*Re*_), at specific resonant frequency, were found for *v* between 0.35 and 0.55. Further addition of turns had negative impact, since extreme values started to decrease and—from an economical point of view—more material was used, enlarging potential cost and mass of the metamaterial. It would be reasonable to prior perform theoretical investigation of potential impact of filling factor on the effective permeability and identify optimum *n* to avoid an unnecessarily large number of turns.

The Eqs. (19) and (20) indicated that uneven absolute values of max and min *μ*_*Re*_ should be observed and both models have shown that asymmetry—the absolute max(*μ*_*Re*_) is higher comparing to min(*μ*_*Re*_). Moreover, these extremes were strongly dependent on the imposed resonant frequency, rather than on filling factor, and this is also in line with theoretical predictions. For identical *v* the optimum min(*μ*_*Re*_) varied from approximately −5 to −25, as shown in Fig. 6b. In other words, the same planar coil will help to achieve not only various resonant frequencies but also effective permeabilities, due to an application of different capacitors.

In the finite grid of resonators each unit cell possesses distinct set of inductive couplings and, resulting from it, sum of mutual inductances, thus resonant frequency and fractional factor will differ within a grid, and therefore each resonator will exhibit various effective permeability at any arbitrary frequency. This explains differences in effective permeabilities, expected and observed, in terms of their distributions on a surface of the physically realized metamaterials. Approach presented in LMM enables to calculate these effective permeabilities for every unit cell independently, by an appropriate application of Eq. (8), i.e., selection of appropriate numbers of rows and columns. Furthermore, it can be also concluded from Fig. 5 that for *f* > > *f*_0_ imaginary part tends to 0, however real part tends to some constant value greater than 0 but less than 1, since based on Eq. (3) we have $\mathop {\lim }\limits_{f \to \infty } \mu_{eff} = 1 - F$. This attribute of metamaterial suggests that it is possible to realize the composite structure with diamagnetic properties, where imaginary parts is negligible and real part of permeability will be approximately constant in some frequency range.

To compare quantitatively numerical (FEM) and analytical model (LMM and LM) a coefficient was proposed, e.g., for LMM given as

22 $$\Delta \mu = \frac{100\% }{5}\sum\limits_{{f_{z} }} {\left[ {\left| {\frac{{\max \left( {\mu_{{\text{Re}}}^{LMM} } \right)}}{{\max \left( {\mu_{{\text{Re}}}^{FEM} } \right)}} - 1} \right| + \left| {\frac{{\min \left( {\mu_{{\text{Re}}}^{LMM} } \right)}}{{\min \left( {\mu_{{\text{Re}}}^{FEM} } \right)}} - 1} \right| + \left| {\frac{{\max \left( {\mu_{{\text{Im}}}^{LMM} } \right)}}{{\max \left( {\mu_{{\text{Im}}}^{FEM} } \right)}} - 1} \right|} \right]} .$$

The coefficient from Eq. (22) expresses the sum of relative differences among extreme values of effective permeability for all five specified resonant frequencies *f*_*z*_. Additionally, to compare errors in estimating the actual resonant frequency similar relative difference was calculated

23 $$\Delta f_{0} = \frac{100\% }{5}\sum\limits_{{f_{z} }} {\left| {\frac{{f_{0}^{LMM} }}{{f_{0}^{FEM} }} - 1} \right|} .$$

**Figure 7:**
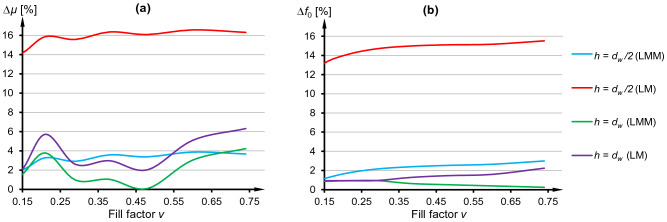
(**a**) Relative difference in extreme values of the effective permeability between analytical and numerical model. (**b**) Relative difference in values of the resonant frequencies between analytical and numerical model.

Cumulative error for LM approach (Δ*μ* ≈ 14 ÷ 17%) was more than four times larger than the one for LMM (Δ*μ* ≈ 2 ÷ 4%) at *h* = *d*_*w*_/2, as it can be seen from Fig. 7a. Relative difference was generally increasing with higher values of *v*. This suggests that the usage of LMM and planar coils with optimum filling factor should bring results to be more accurate. Nevertheless, the differences for LMM and LM for the case with *h* = *d*_*w*_ were smaller (for *v* = 0.48 LMM resulted in nearly 0% error) which can be explained by the decreasing values of *M* with increasing distances between resonators in the periodic grid. In Fig. 7b similar findings can be listed, although LMM and LM for *v* < 0.29 gave comparatively low relative differences in estimating actual resonant frequency. For *v* > 0.29 the differences for LM were increasing but for LMM started to decrease. It was the only discrepancy comparing to Fig. 7a and other results in Fig. 7b.

## Conclusion

In this article the medium frequency metamaterials with vertically stacked multi-turn resonators were presented and analyzed in terms of their computational models and effective properties. Numerical model with homogenized planar resonator and the analytical model, based on Lorentz oscillator, were characterized. The Lorentz model was formulated using electrical lumped parameters of the resonator and a periodic grid, where the mutual inductances between planar coils were taken into account to improve an accuracy of the analytical approach. As a result, the effective permeability may be estimated for any resonators with different number of turns, wires, and sizes of the unit cell, since all lumped parameters have been associated with geometric parameters.

The introduced Lorentz model with mutual couplings eliminates the need of performing numerical computations with the use of 3D model solved in a frequency domain. Hence, the pressure put on memory of a computational unit and the overall solution time can be dramatically reduced. Proposed analytical approach also enables to find the effective permeability of each unit cell in a finite grid, and thus to take into account edge effects appearing in real metamaterials.

An exemplary metamaterial with square planar coils was solved both numerically and analytically. The results showed that by including magnetic couplings of individual resonators in Lorentz model, the differences between numerical and analytical model had been reduced from several to only few percent (or nearly to zero in some cases). While omitting mutual inductances in Lorentz model had led to significant differences in the actual resonant frequency value. Additionally, further analysis had indicated that, in considered metamaterial, it was possible to achieve negative permeability reaching even min(*μ*_*Re*_) = −45, but also the dependency of effective permeability on planar coil filling factor was non-linear, with an optimum value for filling factor between 0.35 and 0.55. The permeability of metamaterial was higher with higher values of the desired resonant frequency; hence it was strongly depending on the capacity of a series connected capacitor. Further research will focus on experimental verification of the abovementioned results, as well as modification of the unit cell geometry, where irregular planar coils will be used.

## Data Availability

The data generated and analysed during the current study is available from the corresponding author.
